# Haemodynamic changes in visceral hybrid repairs of type III and type V thoracoabdominal aortic aneurysms

**DOI:** 10.1038/s41598-023-40323-1

**Published:** 2023-08-23

**Authors:** Chi Wei Ong, Ian J. Y. Wee, Milan Toma, Fangsen Cui, Xiao Yun Xu, Arthur Mark Richards, Hwa Liang Leo, Andrew M. T. L. Choong

**Affiliations:** 1 School of Chemistry, Chemical Engineering and Biotechnology, Nanyang Technological University, Singapore, Singapore; 2 Yong Loo Lin School of Medicine, National University of Singapore, Singapore, Singapore; 3 Department of Osteopathic Manipulative Medicine, College of Osteopathic Medicine, New York Institute of Technology, New York, USA; 4 Institute of High Performance Computing (IHPC), Agency for Science, Technology and Research (A*STAR), Singapore, Singapore; 5 Department of Chemical Engineering, Imperial College London, London, UK; 6 Cardiovascular Research Institute, National University of Singapore, Singapore, Singapore; 7 Christchurch Heart Institute, University of Otago, New Zealand, New Zealand; 8 Department of Biomedical Engineering, National University of Singapore, Singapore, Singapore; 9 Division of Vascular and Endovascular Surgery, Department of Cardiac, Thoracic and Vascular Surgery, National University Heart Centre, Singapore, Singapore; 10 Asian Aortic & Vascular Centre, Singapore, Singapore

**Keywords:** Computational models, Aortic diseases, Biomedical engineering, Computational biophysics

## Abstract

The visceral hybrid procedure combining retrograde visceral bypass grafting and completion endovascular stent grafting is a feasible alternative to conventional open surgical or wholly endovascular repairs of thoracoabdominal aneurysms (TAAA). However, the wide variability in visceral hybrid configurations means that a priori prediction of surgical outcome based on haemodynamic flow profiles such as velocity pattern and wall shear stress post repair remain challenging. We sought to appraise the clinical relevance of computational fluid dynamics (CFD) analyses in the setting of visceral hybrid TAAA repairs. Two patients, one with a type III and the other with a type V TAAA, underwent successful elective and emergency visceral hybrid repairs, respectively. Flow patterns and haemodynamic parameters were analysed using reconstructed pre- and post-operative CT scans. Both type III and type V TAAAs showed highly disturbed flow patterns with varying helicity values preoperatively within their respective aneurysms. Low time-averaged wall shear stress (TAWSS) and high endothelial cell action potential (ECAP) and relative residence time (RRT) associated with thrombogenic susceptibility was observed in the posterior aspect of both TAAAs preoperatively. Despite differing bypass configurations in the elective and emergency repairs, both treatment options appear to improve haemodynamic performance compared to preoperative study. However, we observed reduced TAWSS in the right iliac artery (portending a theoretical risk of future graft and possibly limb thrombosis), after the elective type III visceral hybrid repair, but not the emergency type V repair. We surmise that this difference may be attributed to the higher neo-bifurcation of the aortic stent graft in the type III as compared to the type V repair. Our results demonstrate that CFD can be used in complicated visceral hybrid repair to yield potentially actionable predictive insights with implications on surveillance and enhanced post-operative management, even in patients with complicated geometrical bypass configurations.

## Introduction

Overall treatment for thoracoabdominal aneurysms (TAAA) has improved considerably over the past three decades because of advances in surgical techniques. Although open surgical repair remains popular, it has since been supplanted by total endovascular repair^1,2^. Unfortunately, these operations still carry significant risks of complications and mortality, with the potential of ischemic insult to the spinal cord, kidneys and other visceral organs^3,4^. The visceral hybrid approach^5^ involves surgically rerouting the visceral and renal branches followed by endovascular exclusion of the TAAA with an endograft. This method is an appealing alternative as it avoids the need for thoracotomy or left heart bypass, reduces time under ischemia^6^, and may circumvent the need for catheter manipulations around the renal and mesenteric arteries^7,8^. Early results have proven encouraging although there remains a paucity of data on long-term outcomes in the literature^9^.

Translational research in vascular surgery has offered compelling evidence for a causal association between haemodynamic flow parameters and clinical outcomes. Computational fluid dynamics (CFD) is a useful tool which is gaining traction for its ability to provide a priori predictions concerning the growth or rupture of aortic aneurysms^10^. Indeed, our recent collective study has provided some evidence supporting the application of CFD in clinical studies of aortic aneurysm^11^. Arzani et al. showed that the oscillatory shear index (OSI) is highly associated with thrombus deposition in an abdominal aortic aneurysm^12^. Alterations in the shear stress distribution are also recognized to modulate endothelial cell gene expression^13^, which are in turn linked to increased oxidative stress, reduced nitric oxide production, vessel atherosclerosis, atheroma/neointima formation, aneurysmal growth, and thrombogenic potential^14,15^.

To our knowledge, only one recent CFD analysis has investigated the hemodynamic effect of retrograde visceral revascularization in the abdominal aorta^16^. However, the model may be limited by its simplicity to analyse the complex haemodynamic conditions before and after TAAA therapy. Here, we conducted the first CFD study to explore and compare the haemodynamic changes in two patient-specific TAAAs before and after hybrid repairs with different configurations. Through these simulations, we show that the three-dimensional flow field and changes in wall shear stress can provide important clues to the aetiology of procedural-related complications. The insights gleaned from this analysis could be used to complement pre-operative planning and decision making and provide personalised follow-up and surveillance recommendations for patients in the post-operative setting.

## Methods

### Analysis of pre-operative and post-operative CT scans

**Figure 1:**
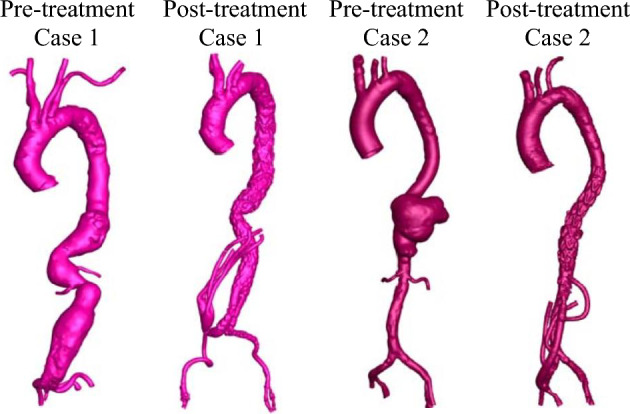
3D patient geometries of two clinical cases were reconstructed from CT images from pre- and post-treatments.

The two cases of TAAA were reconstructed from CT images as showed in Fig. 1. Case 1 is a patient who has been diagnosed with type III TAAA and was scheduled for an elective repair. A four-limbed stent graft was constructed during the procedure. Two inverted (16 × 8 mm) Dacron trouser-grafts function as the conduits. The renal arteries are sequentially anastomosed in an end-to-side fashion. The two remaining graft limbs are routed along the base of the small bowel mesentery to the celiac axis and superior mesenteric artery (SMA) in an end-to-side fashion. The right common iliac artery was used as the inflow. In the second case, the patient had a type V TAAA and underwent emergency repair. Retrograde visceral bypasses (coeliac/SMA) were created from a right common iliac inflow using a bifurcated graft followed by TAAA stent-grafting. However, a type 1b endoleak with associated further aneurysm expansion required a further bifurcated graft to be used for bilateral renal debranching with distal stent graft extension. In both cases, total endovascular aneurysm exclusion was achieved by completion endo-grafting with good results. The study protocol was approved by the Domain Specific Review Board of the National Health Group (Singapore) and written informed consent were obtained from individual patients. All research was performed in accordance with relevant guidelines/regulations.

Geometrical information of thoracic aortic aneurysms was extracted from axial images consisting of 1087–1486 slices (with an in-plane resolution of 512 by 512 pixels and slice thickness of 0.6 mm) obtained from contrast-enhanced CT scans. As depicted in Fig. 1, the images were processed using Mimics, an image processing software (Materialise NV, Leuven, Belgium), to segment the 3D geometries of the aneurysms. The maximum diameters of the aortic aneurysms were measured from the images, with 55.41 mm for case I and 65.89 mm for case II. To ensure smooth and accurate representations, we applied a previously published smoothing approach^17^. Firstly, a mask was created on the CT scans by selecting an appropriate brightness threshold and properly cropping the region of interest to isolate the pure blood flow aortic and vascular lumens. Using this mask, a 3D representation of the lumen surface geometry was computed and then smoothed to eliminate significant variations. Our aim was to preserve the lumen volume, minimize distortion, and optimize the patients’ vessel geometry while keeping computational costs low. We employed two different smoothing algorithms: one with 10 repeats and a high smoothing factor of 0.8, and the other with 30 to 50 repeats and a low smoothing factor of 0.1. The final choice of smoothing parameters was made to achieve minimal model deformation. To ensure the consistency of the smoothing process, the lumen volume was calculated, and a difference of less than 3% before and after the smoothing process was considered acceptable.

### Numerical simulation

**Figure 2:**
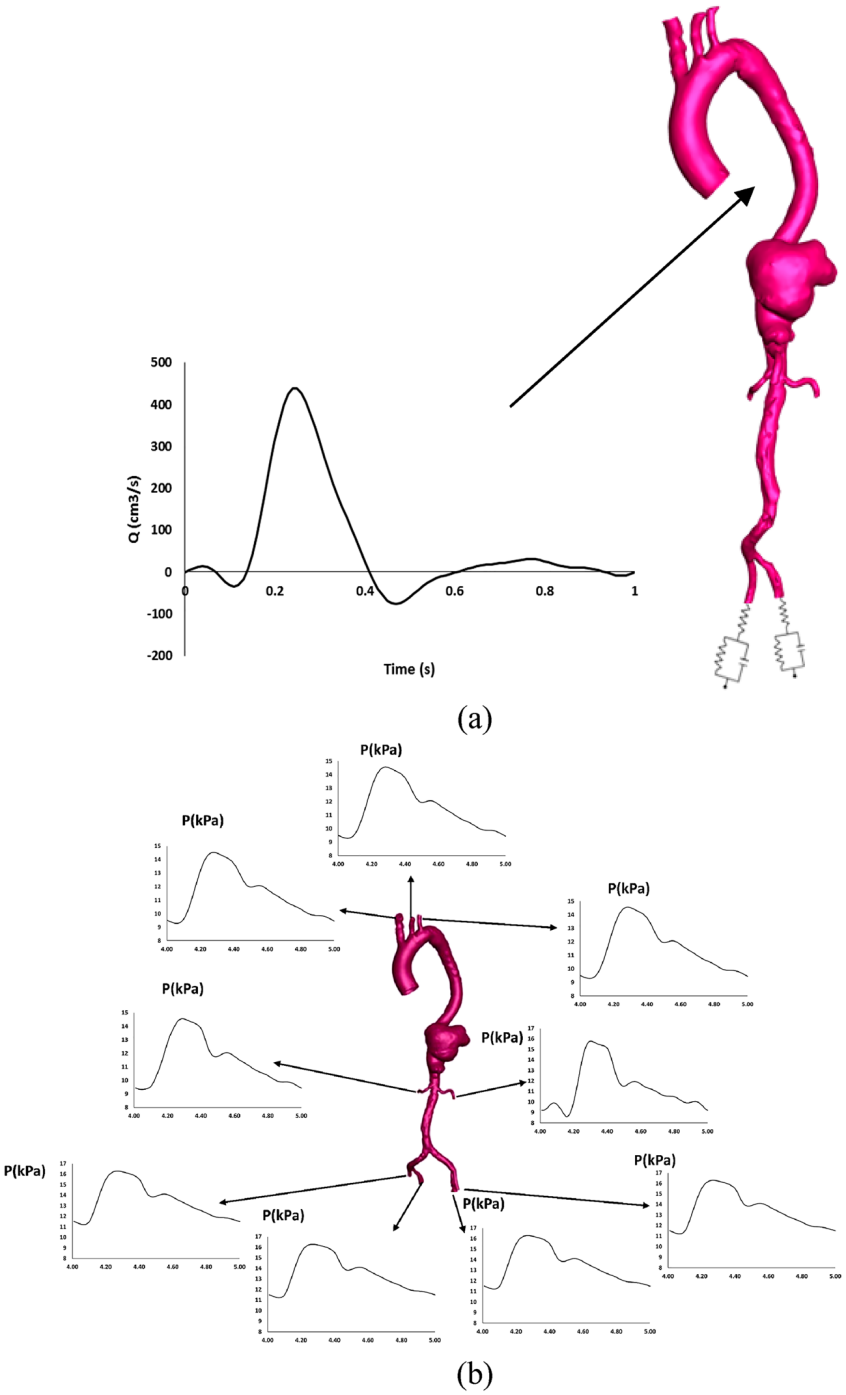
(**a**) Time-dependent inlet velocity profile applied at inlets of all geometries and the three-element Windkessel model was applied at iliac arteries. (**b**) Pressure profile was plotted for each outlet.

Time-dependent transient waveforms of flow rate^18^ were applied at the ascending aorta for all the models (Fig. 2). Based on the literature, the three supra-aortic branches were prescribed 5% of the flow volume^19^ and the renal arteries for the pre-treatment was set to 10% of the thoracic flow^20^. For the post-treatment model, based on the literature, the distributions for the coeliac artery, superior mesenteric artery and renal arteries were set to 31%, 23%, and 23%, respectively^16,21^. For the iliac arteries in the pre and post-TEVAR models, the three-element Windkessel model was adopted as the outlet boundary condition^22–24^. Further details and values of parameters used are provided in the Supplementary Material. The flow rate at the visceral arteries for post-TEVAR was mapped with pressure boundary conditions with a reference to the pressure in a healthy model according to the published literature^16^.

The numerical model was solved in ANSYS FLUENT using the iterative Semi-Implicit Method for Pressure Linked Equations (SIMPLE). A non-Newtonian Carreau fluid model was assumed, as it has been shown to render better modelling of the rheology and viscosity of blood^25^. The graft and arterial wall were set as no-slip conditions. Meshing was done in ANSYS ICEM, and mesh convergence tests were performed to ensure that the numerical calculations are acceptable. The difference in peak wall shear stress during steady flow conditions between different meshing and time steps were within acceptable thresholds (i.e., below 3%)^22^. More details on mesh convergence have been evaluated in Sect. S1 of the Supplementary Text. The transient simulation was run for five cycles with time step set at 0.001 s, and only results from the last cycle will be discussed. We monitored the convergence residuals until the convergence criterion of 10^–5^ was achieved. The Reynolds number is calculated as with a maximum Reynolds number of Re_max_ = 1725 among all the cases.

To examine the alterations in helical flow before and after visceral repair, we utilized helicity as a quantifying parameter. Helicity was determined by calculating the absolute local normalized helicity as Eq. (1)

1 $$Helicity= \frac{\mathrm{V}\cdot\upomega }{\left|\mathrm{V}\right|\left|\upomega \right|},$$

where V represents the velocity field, and ω is the vorticity determined through ∇ × V^26–28^. Helicity serves as a directionally independent voxel-wise measure, with a value of 0 denoting no helical flow and a value of 1 denoting maximal helical flow. We evaluated the risk of thrombosis by calculating the time-averaged wall shear stress (TAWSS) and endothelial cell activation potential (ECAP). TAWSS is defined as the averaged WSS acting upon the wall for the entire cardiac cycle^29^. The oscillatory shear index (OSI) describes the oscillation of the wall shear stress^30^. Endothelial cell activation potential (ECAP)derived as the quotient of OSI and TAWSS has been shown to characterize the ‘thrombogenic susceptibility’ of the vessel wall^31^. The relative residence time (RRT) is a frequently used metric for estimating the duration that particles stay in close proximity to the wall^32,33^. RRT is calculated using the Eq. (2)

2 $$RRT=\frac{1}{\left(1-2\times OSI\right)\times TAWSS},$$

where OSI refers to oscillatory shear index and TAWSS represents time-averaged wall shear stress. This formula allows for the quantification of the residence time and provides insights into the hemodynamic conditions that influence particle–wall interactions.

### Informed consent statement

Informed consent was obtained from all subjects involved in the study.

## Results

The haemodynamic changes of flow patterns for both treated and untreated TAAA were separately investigated at four phases of the cardiac cycle (early systole, mid systole, late systole and early diastole).

**Figure 3:**
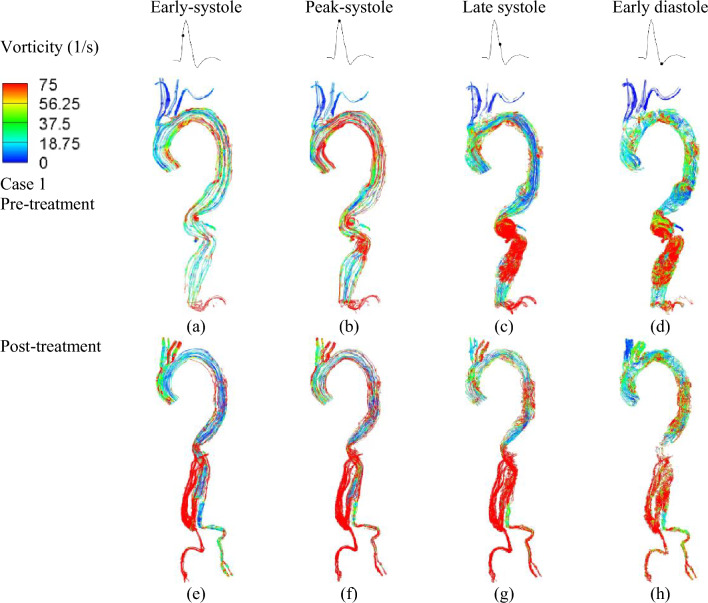
Instantaneous vorticity plots were generated at four representative time instants. Top row: vorticity changes of Type III TAAA before treatment at different time instants in the cardiac cycle: (**a**) early systole, (**b**) peak systole, (**c**) late systole, and (**d**) early diastole. Bottom row: vorticity changes after post-treatment at different time instants in one cardiac cycle: (**e**) early systole, (**f**) peak systole, (**g**) late systole, and (**h**) early diastole.

The vorticity plots obtained from the patient-specific simulations are summarized below. Differences in vorticity dynamics were observed between the two pre- and post-treatment cases. In Case I, which involves type III TAAA, the presence of a high curvature at the inner wall near the abdominal region, combined with rapid systolic forward flow, resulted in the development of elevated vorticity in this inner wall region, forming a shear layer (Fig. 3a–d). During late systole, retrograde flow emerged in the region near the anterior wall of the proximal descending aorta, creating a highly recirculating region with intense vorticity magnitude. However, this intense region was resolved by the visceral hybrid repair, as no high vorticity was observed along the abdominal region, except for a small region at the right iliac artery (Fig. 3e–h).

**Figure 4:**
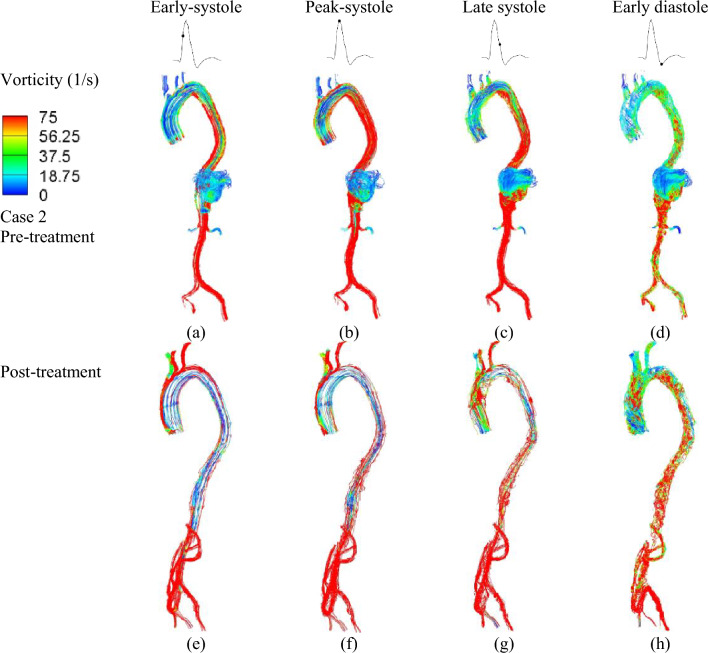
Instantaneous vorticity plots were generated at four representative time instants. Top row: Vorticity changes of Type V TAAA before treatment at different phases in the cardiac cycle: (**a**) early systole, (**b**) peak systole, (**c**) late systole, and (**d**) early diastole. Bottom row: Vorticity changes after treatment at different time instants in one cardiac cycle: (**e**) early systole, (**f**) peak systole, (**g**) late systole, and (**h**) early diastole.

In Case II, involving type V TAAA, the aneurysm is located outside the main aorta region and exhibits a less symmetric geometry compared to type III TAAA. The combination of fast-moving forward flow and the luminal expansion of the aneurysm creates a highly disturbed flow region that persists throughout the entire cardiac cycle. The presence of retrograde flow from the distal end of the aorta further amplifies the overall vorticity and leads to the formation of a recirculation region within the aneurysmal area (Fig. 4a–d). Another noteworthy observation is that during the late systole to early diastole phase, these regions of weak vorticity can cause the release of blood particles from within the vortex, particularly at the anterior portion of the vortex. These particles then flow towards the posterior wall of the aneurysm, resulting in the formation of a stagnation flow region. However, this stagnation flow region is subsequently resolved by the visceral hybrid repair (Fig. 4e–h).

**Figure 5:**
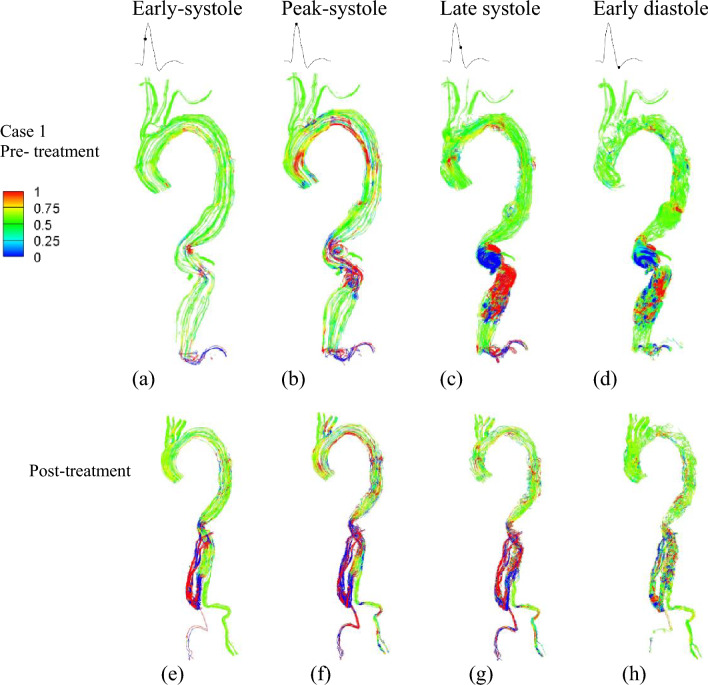
Helicity was plotted at four representative time instants. The top row illustrates variations of helicity in pre-treatment Type III TAAA at: (**a**) early systole, (**b**) peak systole, (**c**) late systole, and (**d**) early diastole. The bottom row displays variations of helicity after treatment at: (**e**) early systole, (**f**) peak systole, (**g**) late systole, and (**h**) early diastole.

**Figure 6:**
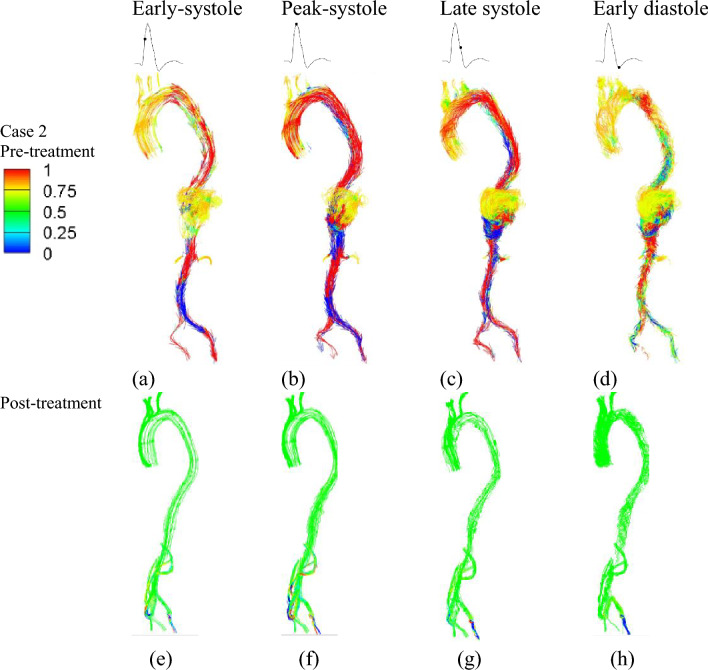
Helicity was plotted at four representative time instants. The top row illustrates variations of helicity in pre-treatment Type V TAAA at: (**a**) early systole, (**b**) peak systole, (**c**) late systole, and (**d**) early diastole. The bottom row displays variations of helicity after treatment at: (**e**) early systole, (**f**) peak systole, (**g**) late systole, and (**h**) early diastole.

Helical flow patterns are prominently observed during systole in both cases before treatment, as highlighted by the computational results of helicity presented in Figs. 5 and 6. In the case of type III TAAA, the flow exhibits a dominant rotational nature throughout the systole (Fig. 5a–d). This results in the emergence of a numerically large helicity value in the lumen near the abdominal region, which subsequently gets redistributed through the four-limbed stent graft in the visceral hybrid repair (Fig. 5e–h). Furthermore, we observed that these high helicity flow features are effectively reduced during the early diastole (Fig. 5d). Additionally, we noticed relatively low helicity in the right iliac artery, which can be attributed to the sharp bending of the stent graft configuration.

For type V TAAA, we observed a decrease in helicity flow within the aneurysm, primarily attributable to its highly asymmetric geometry (Fig. 6a–d). After the visceral hybrid repair, the average helicity decreased further. Unlike the case of type III TAAA visceral repair, where prominent helical flow was observed in the four-limbed stent graft, no significant helical flow patterns were observed in the corresponding regions of the type V TAA visceral hybrid repair (Fig. 6e–h). However, it is noteworthy that the iliac artery exhibited higher helicity after the graft deployment, regardless of whether it was in an antegrade or retrograde direction.

**Figure 7:**
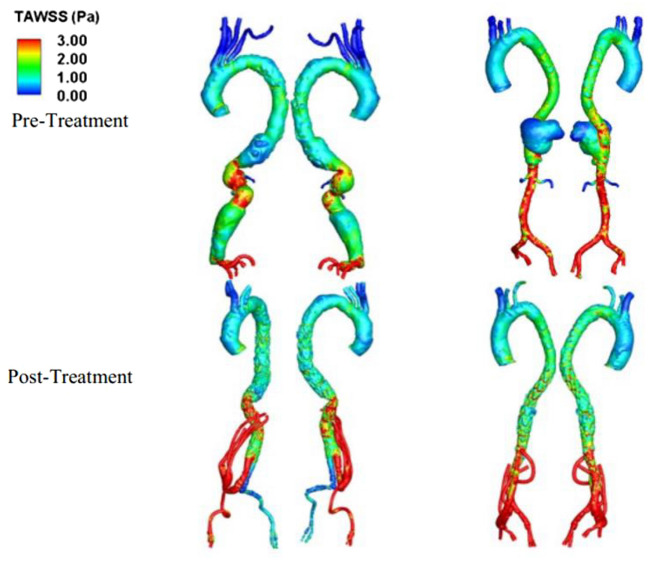
Time-averaged wall shear stress (TAWSS) distribution for pre- and post-treatment. Left: case 1 (elective repair), right: case 2 (emergency repair).

Figure 7 presents the TAWSS for both clinical cases before and after treatment. We found low TAWSS (< 1 Pa) at the posterior side of aneurysm in case 1, which was eliminated after treatment. For case 2, we observed low TAWSS at the posterior side of the aneurysm. However, there was no low TAWSS at the entry of the stent graft and the right common iliac artery. High TAWSS (> 3 Pa) was found at the placement of the stent graft in both cases.

**Figure 8:**
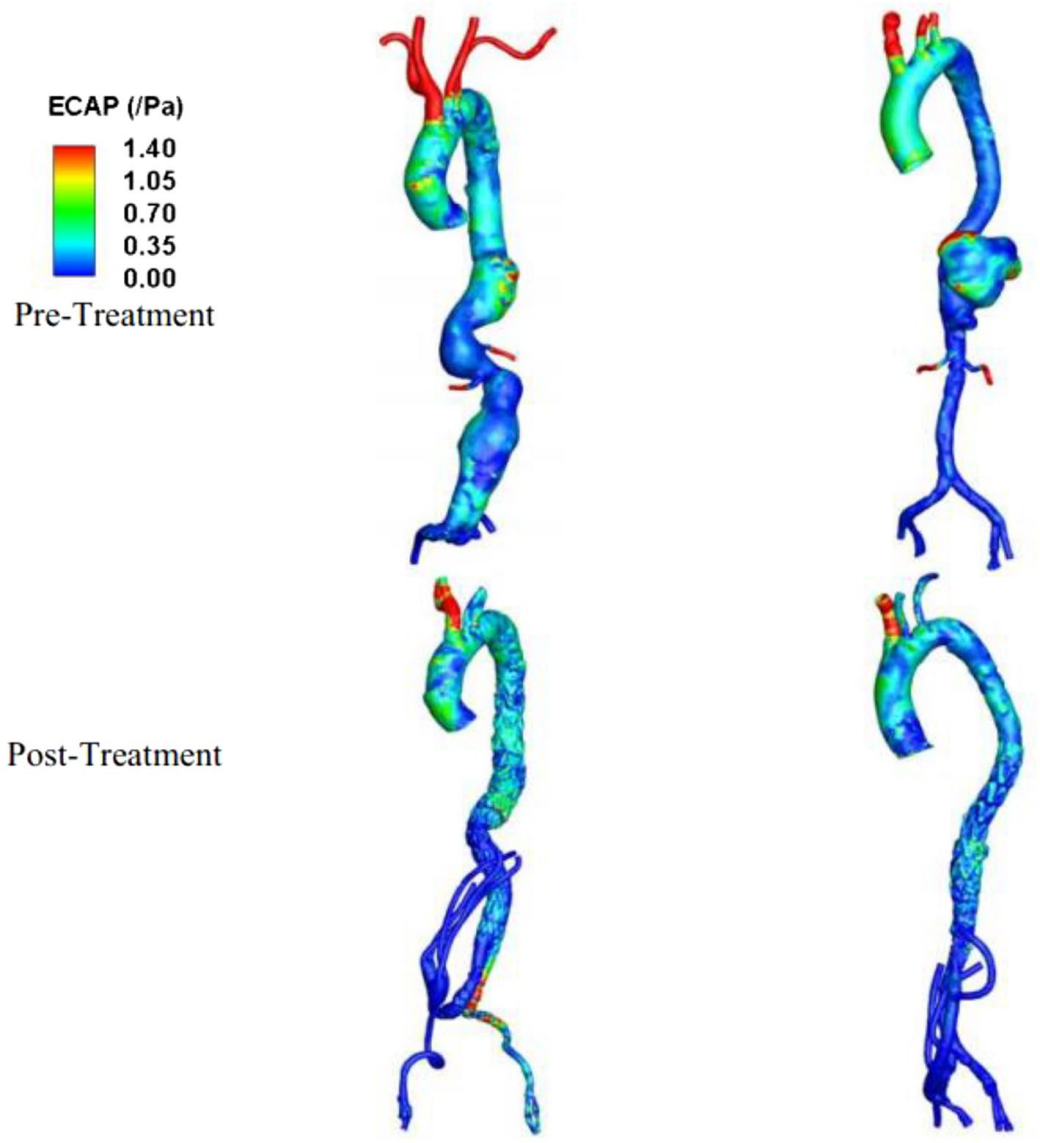
Spatial distribution of endothelial cell action potential (ECAP) for the pre- and post-treatment for two different configurations of treatment. Left: case 1 (elective repair), right: case 2 (emergency repair).

Endothelial cell action potential (ECAP) was evaluated as shown in Fig. 8. Before treatment, the TAAA in case 1 had a small region with high ECAP (> 1.4 Pa^−1^) at the posterior side of the aneurysm, which was reduced post-treatment. This was accompanied by a high ECAP at the right common iliac artery. Case 2 had a large region exposed to high ECAP at the anterior side of aneurysm, which was eliminated post-treatment.

**Figure 9:**
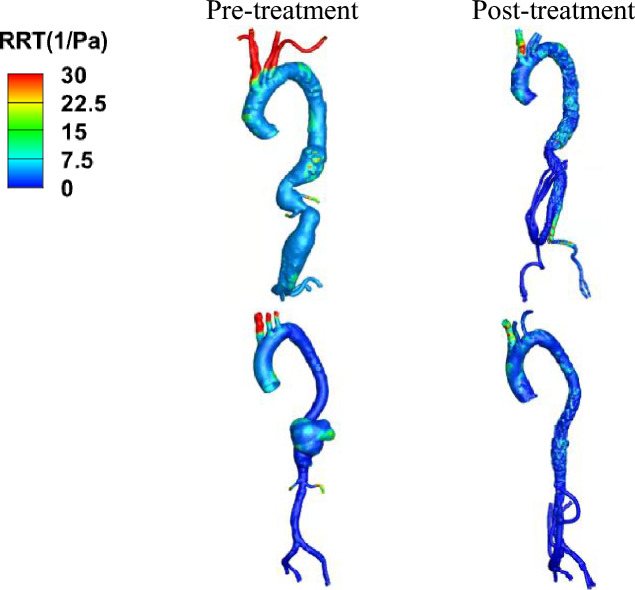
Spatial distribution of relative residence time (RRT) for the pre- and post-treatment for two different configurations of treatment. Upper row: case 1 type III TAAA before and after treatment, bottom row: case 2 type V TAAA before and after treatment.

Results for RRT are shown in Fig. 9. Prior to treatment, a region of elevated RRT (> 5 s) was observed within the aneurysm for type III TAAA. Similarly, high RRT values were observed in type V TAAA before the visceral repair. However, following the visceral repair, the high RRT region was significantly reduced. Nevertheless, in type III TAAA, a region exposed to elevated RRT persisted after the visceral repair due to the configuration of the four-limbed stent graft, which aligns with the presence of the high ECAP region.

## Discussion

To the best of our knowledge, this study is the first to explore haemodynamic differences before and after two different visceral hybrid repairs for thoracoabdominal aneurysms with complicated geometrical features using patient-specific images. A comprehensive analysis was conducted on hemodynamics in pre and post-operative conditions by examining vorticity, helicity, time-averaged wall shear stress (TAWSS), relative residence time (RRT) and endothelial cell action potential (ECAP). The results of our study provide insights into the impact of different surgical approaches on hemodynamic parameters in patients with complex thoracoabdominal aneurysms. These findings can contribute to our understanding of the clinical outcomes associated with these procedures and may assist in informing surgical decision.

The quantification of helicity measures the degree of rotation and spiral movement within a fluid flow. High helicity is considered to have favorable physiological effects as it promotes efficient transportation of blood, reduces flow disruptions, and helps prevent the accumulation of atherogenic particles on arterial surfaces^26,34–36^. In this study, helicity was utilized to quantitatively differentiate flow patterns in various visceral repair configurations, along with the analysis of vorticity features and wall shear stress parameters. The findings of the study suggest that the hybrid repair approach utilizing an endovascular stent graft and open visceral reconstruction may offer advantages over a total endovascular approach in terms of hemodynamic performance in selected patients. In addition, published research suggests that blood flow in more tortuous vessels exhibits higher helicity^26,27,36,37^. In our observations during the pre-operative stage of Case 1 and Case 2, we found a significant difference in helicity magnitude in the “non-pathological” thoracic portion of the vessel. The relatively higher helicity observed in the pre-operative stage of Case 2 in the ascending aorta, compared to Case 1, can be attributed to the more tortuous nature of the region, which can also mitigate flow distortion. This finding is consistent with existing literature^26^. Flow patterns are closely related to the anatomical features of the aneurysm before treatment. Changes in flow patterns can affect thrombus formation. The deposition of platelets on the aneurysmal wall is associated with the recirculation flow and helicity, which can ultimately lead to thrombosis^38,39^. Kelsey et al. showed that ECAP > 1.4 Pa are found at the thrombus site^31^ which can be used as a critical threshold to evaluate the chance of thrombus^40^. Low TAWSS with high ECAP and high RRT is also correlated to the regions of intraluminal thrombus development^41,42^. As observed in the vorticity of case 1 in Fig. 3, the tortuous geometry changes the results of flow direction in the recirculation region with stagnation flow found at the posterior. The low vorticity caused low TAWSS at the posterior side of the aneurysm with a high ECAP value (> 1.4 Pa^−1^). The recirculation region was found to occupy the entire aneurysm in case 2 throughout the cardiac cycle. The occurrence of such recirculation zones with low vorticity (< 18.75 s^−1^) breaks into a disturbed flow region with low helicity in early diastole, which can redistribute the platelets to regions of low TAWSS, thereby initiating the process of the thrombus formation. This corresponds to a higher ECAP (> 1.4 Pa^−1^) and high RRT (> 5 s) at the posterior side of the aneurysm, which can indicate thrombus formation in the aneurysm. Our results indicate that the presence of an aneurysm can alter the vorticity and helicity within the blood vessels, potentially leading to an increased risk of blood clots, thrombosis, or other complications. It is important for doctors to carefully evaluate the flow patterns within an aneurysm before deciding on a course of treatment, in order to ensure the best possible outcome for the patient.

Retrograde flow in the bypass grafts following a visceral hybrid procedure has been previously studied in an idealized model but not in a patient-specific model^16^. In our patient-specific studies, we found that in the elective repair for case 1, in which the neo-bifurcation of stent graft is high, the right iliac artery was exposed to an increased risk of thrombosis evident from the high ECAP value (1.4 Pa^−1^) and high RRT value (> 5 s) accompanied by an extremely low TAWSS (< 0.3 Pa) with low helicity due to the stent graft configuration. We have further shown that by changing the configuration, the risk of thrombosis can be reduced, as demonstrated in Sect. S4 of the supplementary text with a reduced ECAP value. No high ECAP region was found postoperatively in Case 2. We hypothesize that in Case 1, the high ECAP and high RRT found in post operative case was due to the large volume of flow exiting the left iliac artery through the stent graft. Helical flow is believed to have a protective role against atherosclerosis in the circulatory system. However, the use of a four-limbed stent graft configuration may diminish this protective effect and expose the iliac arteries to a more pathological condition in the post-operative phase. This visceral hybrid repair may create a less physiological flow structure compared to a healthy aorta. In a healthy aorta, helical flow contributes to improved blood flow efficiency, especially in the presence of distorted arterial geometry^26^. Our findings from the two different visceral repairs demonstrate that the implementation of a four-limbed stent graft configuration could attenuate this effect but paradoxically exacerbate the adverse consequences associated with the distributed flow region.

Clinically, the occurrence of retrograde flow in the bypass graft can induce blood flow disturbances, such as recirculation and the subsequent trapping of particles at inflow sites. This could lead to in-graft failure or unfavourable stent patency^16,43^. Several steps can be taken to prevent in-graft failure or unfavourable stent patency. First, it is important to carefully select the appropriate type of stent according to the specific application. Second, the stent should be properly sized and positioned to ensure proper fit and function. Third, the stent should be securely attached to the vessel to prevent it from moving or becoming dislodged. Therefore, in this case, it is important to closely monitor the stent post-implantation to ensure that it is functioning properly and to promptly address any issues that may arise.

This is a pilot study to describe the haemodynamic changes between pre- and post-visceral hybrid repairs of thoracoabdominal aneurysms. The main limitation is the use of only two sets of CT images without continuous follow-up studies to confirm our predicted risk of thrombus after hybrid treatment. We acknowledge that using literature-based velocity and pressure profiles is a limitation in the present study. Furthermore, it is possible that wall shear stress values have been overestimated by the CFD model employed herein. As such, conducting an FSI investigation could yield more precise outcomes for future research endeavours. Other potential areas of improvement may involve enlisting a larger patient sample with personalized boundary conditions derived from both initial examinations and follow-up assessments to enhance accuracy.

## Conclusion

Using computational fluid dynamics, we present a unique preliminary study of two different patient-specific haemodynamic patterns for geometric configurations of visceral hybrid repairs (retrograde visceral and renal bypass grafts and completion endovascular stent grafting) of thoracoabdominal aortic aneurysms. The findings suggest that an image-based CFD study can potentially be used to predict the risk of thrombus formation for visceral hybrid repair. Our data suggests a potential predictive role of CFD in complex aneurysm repair, with implications for post-operative surveillance. Future research can further investigate the role of CFD in predicting post-operative outcomes in visceral hybrid repairs.

### Supplementary Information

Supplementary Information.

## Data Availability

The data are not publicly available as this may compromise the individual privacy. Data may be available from the corresponding author upon reasonable request.
