# Supplementary material for: Haemodynamic changes in visceral hybrid repairs of type III and type V thoracoabdominal aortic aneurysms
Source: Sci Rep. 2023 Aug 23;13:13760. doi: 10.1038/s41598-023-40323-1 (PMC10447573; doi:10.1038/s41598-023-40323-1)
Supplement: Supplementary file 1 — Supplementary Information. [file 41598_2023_40323_MOESM1_ESM.docx]

Supplementary Material

**S1 Mesh convergence test**

The models presented in the current study were meshed using ICEM 16 (Ansys Inc.), and mesh sensitivity tests were conducted to ensure mesh independence for each scenario, following the approach described in the published literature [1]. The primary objective of the sensitivity test was to establish mesh independence of the flow field, which has an impact on thrombus formation. For each geometric model, three different meshes were examined based on an element characteristic length ranging from 0.5 to 3 mm [2]. It was deemed satisfactory if the discrepancies in peak wall shear stress were below 5%. Mesh convergence data was presented from supplementary tables 1 to 4, with the bold one being the final mesh we selected. The final grids were selected to strike a balance between accuracy and computational cost and consisted of approximately 314,893 to 35,831,604 elements with a tetrahedral core and 10 prismatic layers near the walls.

Simulations were conducted using a workstation equipped with an Intel Xeon E5-2699 v4 2.20 GHz processor, featuring 22 cores, 55M Cache, and 128 GB DDR4 RAM. Each simulation took approximately 240 hours per cardiac cycle. To ensure accuracy and reliability, we performed 5 cycles for each model, with the results from the final cycle being presented in this study. The time for one simulation took up to 240 hours because the computer had to solve >10 million equations per iteration for pulsatile flow motion.

**Supplementary table 1.** Mesh convergence data for type III TAAA Pre-treatment

|  | **Mesh type** | **Minimum Size** | **Maximum Size** | **Number of elements** | **Area-averaged TAWSS(Pa)** |
| --- | --- | --- | --- | --- | --- |
|  | Coarse mesh | 0.4 | 2 | 3148932 | 0.831498 |
|  | Fine mesh | 0.5 | 1.8 | 4397392 | 0.838031 |
|  | **Finer mesh** | **0.5** | **1.6** | **12337707** | **0.834849** |

**Supplementary table 2.** Mesh convergence data for type III TAAA Post-treatment

| **Mesh type** | **Minimum Size** | **Maximum Size** | **Number of elements** | **Area-averaged TAWSS (Pa)** |
| --- | --- | --- | --- | --- |
| Coarse mesh | 0.4 | 2 | 5767544 | 0.819249 |
| Fine mesh | 0.5 | 1.8 | 7235218 | 0.816962 |
| **Finer mesh** | **0.5** | **1.6** | **13511287** | **0.820328** |

**Supplementary table 3.** Mesh convergence data for type V TAAA Pre-treatment

|  | **Mesh type** | **Minimum Size** | **Maximum Size** | **Number of elements** | **Area-averaged TAWSS(Pa)** |
| --- | --- | --- | --- | --- | --- |
|  | Coarse mesh | 0.4 | 2 | 7066963 | 0.845043 |
|  | Fine mesh | 0.5 | 1.8 | 12863323 | 0.853663 |
|  | **Finer mesh** | **0.5** | **1.6** | **35831604** | **0.858877** |

**Supplementary table 4.** Mesh convergence data for type V TAAA Post-treatment

|  | **Mesh type** | **Minimum Size** | **Maximum Size** | **Number of elements** | **Area-averaged TAWSS(Pa)** |
| --- | --- | --- | --- | --- | --- |
|  | Coarse mesh | 0.4 | 2 | 4484241 | 0.84715 |
|  | Fine mesh | 0.5 | 1.8 | 6250474 | 0.849293 |
|  | **Finer mesh** | **0.5** | **1.6** | **11710798** | **0.860391** |

**S2 Windkessel Model**

The Windkessel model value parameters we use at the iliac outlet are listed in supplementary table 5. It is referenced in the following source [3].

**Supplementary table 5.** Windkessel model parameters and value

| Parameter | Value |
| --- | --- |
| Windkessel resistance R_1_ | 1.1752 · 10^7^ Pa s m^−3^ |
| Windkessel compliance, C | 1.0163 · 10^8^ Pa s m^−3^ |
| Windkessel resistance, R_2_ | 1.1167 · 10^8^ Pa s m^−3^ |

References

[1] Menichini, C., Cheng, Z., Gibbs, R. G., & Xu, X. Y. (2018). A computational model for false lumen thrombosis in type B aortic dissection following thoracic endovascular repair. Journal of biomechanics, 66, 36-43.

[2] Sotelo, J., Urbina, J., Valverde, I., Tejos, C., Irarrázaval, P., Andia, M. E., ... & Hurtado, D. E. (2016). 3D quantification of wall shear stress and oscillatory shear index using a finite-element method in 3D CINE PC-MRI data of the thoracic aorta. IEEE transactions on medical imaging, 35(6), 1475-1487.

[3] Xiao, N., Alastruey, J., & Alberto Figueroa, C. (2014). A systematic comparison between 1‐D and 3‐D hemodynamics in compliant arterial models. International journal for numerical methods in biomedical engineering, 30(2), 204-231.

**S3 Pressure distribution**

The pressure distribution analysis demonstrates that the visceral repair leads to an improved pressure distribution compared to the pre-treatment condition, as illustrated in the supplementary figure 1.

|  | Pre-treatment | Post-treatment |
| --- | --- | --- |
| Pressure (Pa)  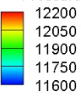  Case I Type III TAAA | 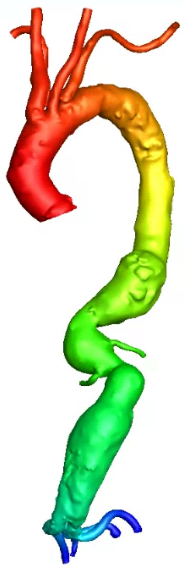 | 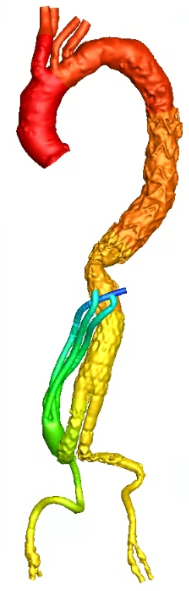 |
| Case 2 Type V TAAA | 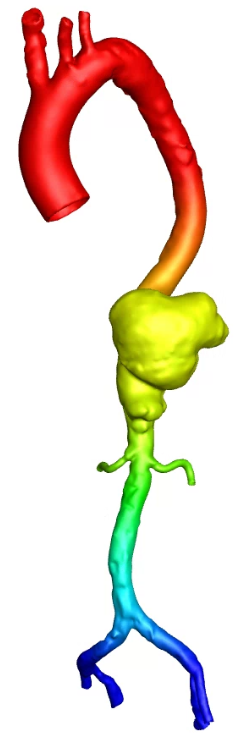 | 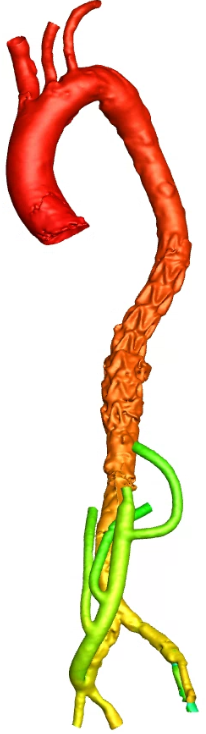 |

**Supplementary figure 1.** Pressure distribution for the pre- and post-treatment for two different configurations of treatment. Upper row: case 1 type III TAAA before and after treatment, bottom row: case 2 type V TAAA before and after treatment

**S4 Hemodynamic change under different stent graft configuration**

We made modifications to the *in-silico* model by adjusting the stent graft configuration of the right iliac artery from 90 degrees to 0 degrees. Our findings revealed a significant reduction in the region exposed to high endothelial cell action potential (ECAP) when the bending angle of the stent graft configuration was modified as shown in supplementary figure 2. This change in the bending angle of the stent graft configuration also promotes a smoother flow, minimizing the occurrence of eddy flows in the iliac artery compared to the original configuration.

| 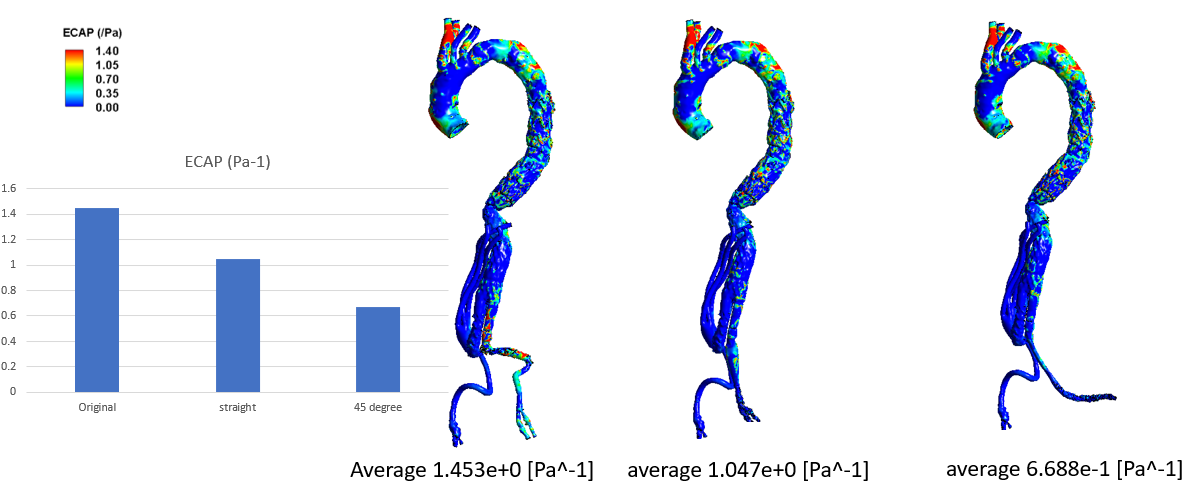 | 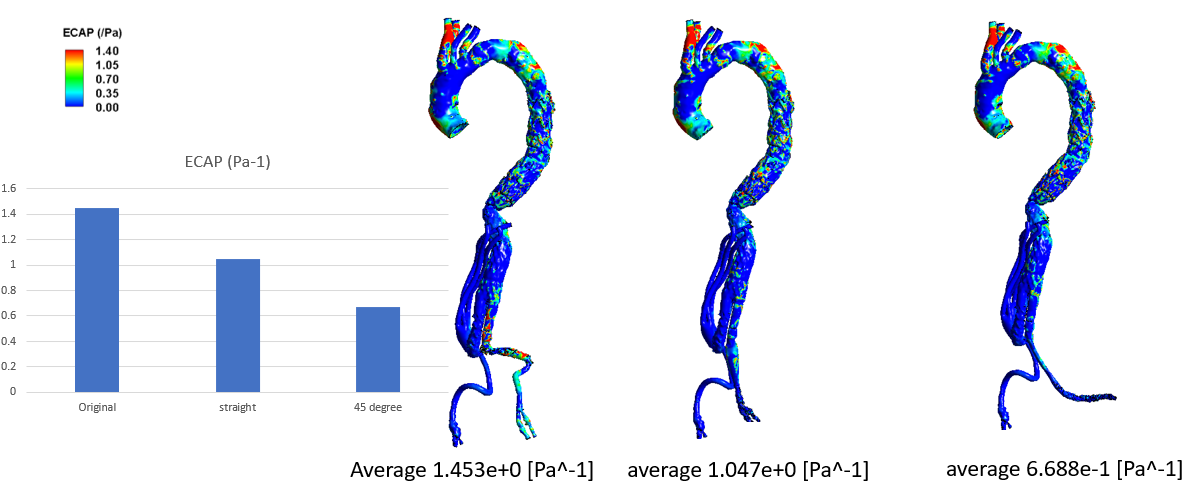 | 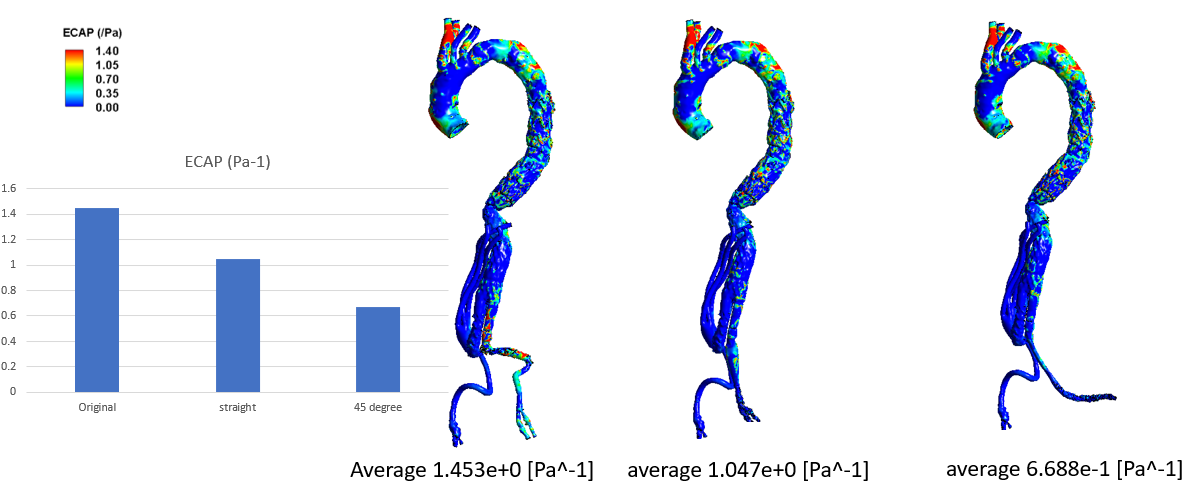 | 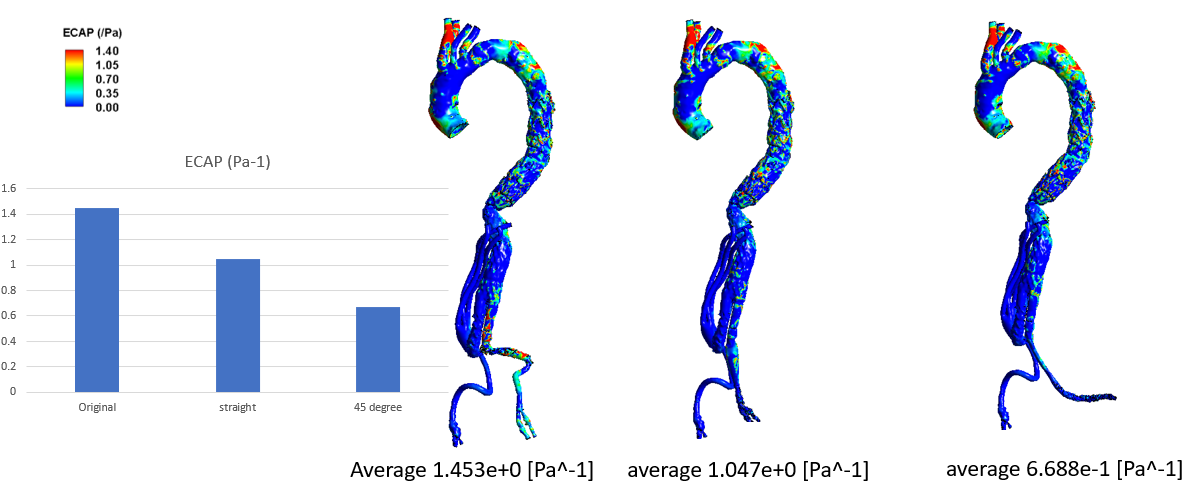 |
| --- | --- | --- | --- |
|  | (a) | (b) | (c) |

**Supplementary figure 2.** Spatial distribution of endothelial cell action potential (ECAP) for three different configurations of treatment. (a) 90 degree (Original) (b) 0 degree (c) 45 degree
